# COVID-19 vaccination motivation and underlying believing processes: A comparison study between individuals with affective disorder and healthy controls

**DOI:** 10.3389/fpsyg.2022.935278

**Published:** 2022-12-06

**Authors:** Nina Dalkner, Eva Fleischmann, Frederike T. Fellendorf, Jolana Wagner-Skacel, Elena M. D. Schönthaler, Susanne Bengesser, Alfred Häussl, Sophie Tietz, Adelina Tmava-Berisha, Melanie Lenger, Eva Z. Reininghaus

**Affiliations:** ^1^Clinical Department of Psychiatry and Psychotherapeutic Medicine, Medical University Graz, Graz, Austria; ^2^Clinical Department of Medical Psychology, Psychosomatic, and Psychotherapy, Medical University Graz, Graz, Austria

**Keywords:** COVID-19 vaccination, affective disorder, cognition, emotion, credition

## Abstract

**Background:**

Believing processes represent fundamental brain functions between cognition and emotion. Shortly before the introduction of a compulsory vaccination against COVID-19 in Austria, motives and underlying believing processes regarding the vaccination were collected in individuals with affective disorder (AD) and healthy controls (HC).

**Methods:**

79 individuals with AD and 173 HC were surveyed online to assess believing processes with the parameters of the credition model (narratives, certainty, emotion, mightiness) about (1) the coronavirus itself and (2) why someone is vaccinated or not. In addition, we calculated congruence scores between content of narrative and type of emotion and divided the narrative content into positive, negative, and indifferent.

**Results:**

There were no differences in vaccination status between AD and HC. Higher levels of certainty were observed in HC compared to AD in both vaccinated and unvaccinated individuals. The effects were higher when asked about the motivation to vaccinate or not than about the coronavirus itself. In HC, more positive emotions and more congruence between emotions and narratives were reported during believing in their vaccination motives. No group differences were found in mightiness for both items. Independently from diagnosis, unvaccinated people had high levels of certainty and more negative emotions and narratives while believing in their motives for not getting vaccinated.

**Conclusion:**

When believing about the COVID-19 vaccination, individuals with AD were more uncertain and experienced fewer positive emotions than HC, although both groups did not differ in vaccination status. These effects were not that strong when believing about the coronavirus in general.

## Introduction

In Austria, containment measures against the coronavirus disease (COVID)-19 issued by the government included the obligation to be vaccinated or recovered when in public. In the period from December 1, 2021, to January 31, 2022, the number of individuals tested positive for COVID-19 increased from 1,175,785 to 1,891,468, and the number of deaths from or with COVID-19 increased from 12,458 to 13,669 (AGES, 2022; Epidemiologisches Meldesystem, 2022b). Starting on November 15, 2021, a lockdown for unvaccinated individuals was introduced, which lasted until January 31, 2022 (Niederösterreichische Nachrichten, 2022). A general lockdown was imposed from November 22 to December 11, 2021. On January 11, 2022, the decision of a nationwide vaccination obligation was proclaimed and with February 5, 2022, compulsory COVID-19 vaccination was required for adults aged 18 and older (Bundesministerium für Soziales, Gesundheit, Pflege und Konsumentenschutz, 2022a), which has been suspended again since March 9_,_ 2022 (Bundesministerium für Soziales, Gesundheit, Pflege und Konsumentenschutz, 2022b). Up until January 31, 2022, 75.9% of Austrians had been vaccinated at least once, 72.1% had been vaccinated twice, and 49.8% had received the third shot (Epidemiologisches Meldesystem, 2022a). In comparison, 63.3% of Europeans had been vaccinated two times (Our World in Data, 2022).

Vaccination rates of individuals with psychiatric disorder were lower than those of the general population, despite having been given priority status in some countries (Tzur Bitan et al., 2021; Arumuham et al., 2022; Curtis et al., 2022). One reason for this might be vaccine hesitancy, which was more pronounced in individuals with mental illness than in healthy controls (HC; Hao et al., 2021; Jefsen et al., 2021; Eyllon et al., 2022). Factors associated with vaccine hesitancy were misinformation, fear (Payberah et al., 2022; Peritogiannis et al., 2022), mistrust (Payberah et al., 2022), and negative attitudes towards vaccines (Danenberg et al., 2021). Believing in the safety of vaccines and a good preventive effect were associated with vaccination willingness in individuals with psychiatric disorder (Huang et al., 2021).

Believing is a cognitive process consisting of formation, revision, and evaluation of beliefs (Angel and Seitz, 2016; Connors and Halligan, 2017). Credition describes the dynamic process of believing (Angel, 2013; Paloutzian and Mukai, 2017) as an interface between cognition and emotion. The credition model by Angel and Seitz (2016) encompasses four major parameters: proposition, certainty, emotion, and mightiness. The content of the statement about a certain belief is called “proposition.” A person’s inclination to believe the proposition is referred to as “certainty.” The affective valence of the proposition is termed “emotion.” The degree of significance of the proposition is termed “mightiness.”

In a recent study during the COVID-19 pandemic, our study group demonstrated that credition parameters highly differed between patients with bipolar disorder and HC (Tietz et al., 2022). As the attitude towards the COVID-19 vaccination and motives to get vaccinated of individuals with psychiatric disorder remain largely unexplored and the underlying cognitive processes are unknown, we aimed to investigate believing processes around COVID-19 vaccination and to compare patients with affective disorders (AD) and HC. Additionally, we aimed to test for differences in believing depending on vaccination status (vaccinated or not vaccinated), as the understanding of believing processes (narrative, certainty, emotion, and mightiness) can provide a better overview of the motivators for vaccination and consequently increase the vaccination rate of people who are particularly at risk.

## Materials and methods

An online survey was conducted with LimeSurvey (GmbH, 2003) and a link was sent out *via* e-mail to a pool of currently and previously treated patients at the Department of Psychiatry and Psychotherapeutic Medicine in Graz and was also shared *via* social media. The survey took place from December 14, 2021 to January 31, 2022. The study was approved by the local ethics committee and informed consent was given prior to study participation. In sum, 356 people opened the survey (104 of them indicated having a psychiatric disorder), and 252 (79 AD and 173 HC) of them filled out all items and were included in the analyses. The participants were surveyed on their vaccination status, demographic data, and with two questions in German language concerning their individual beliefs. The items of interest are listed in Table 1.

**Table 1:** Items of interest (Believing processes, vaccination status, psychiatric diagnosis control items).

**Believing processes** *Item 1 COVID-19 beliefs:* *a*. Proposition: *When I think about the coronavirus (COVID-19), I believe that … (narrative)* *b*. Certainty: *On a scale from 0 (not sure) to 100 (quite sure), how sure are you about that while believing?* *c*. Emotion: *Using the Emotion Wheel, please identify an emotion that most closely relates to your state while you are believing:…* *d*. Mightiness: *On a scale of 0 (not at all) to 10 (very much), how strongly do you experience the emotion while believing?* *Item 2 Vaccination/Non-vaccination motive beliefs:* *a*. Proposition: *I am vaccinated/not vaccinated against COVID-19, because I believe that … (narrative)* *b*. Certainty: *On a scale from 0 (not sure) to 100 (quite sure), how sure are you about you about that while believing?* *c*. Emotion: *Using the Emotion Wheel, please identify an emotion that most closely relates to your state while you are believing:…* *d*.Mightiness: *On a scale of 0 (not at all) to 10 (very much), how strongly do you experience the emotion while believing?* **Vaccination status** *Have you been vaccinated against COVID-19? [Yes, fully immunized (at least 2 vaccinations)/Yes, one vaccination/No]* **Psychiatric Diagnosis Control Items** 1. *Please indicate which psychiatric disorder(s) you currently have (multiple answers possible): [None/Depressive disorder/Bipolar disorder/Panic disorder/Generalized Anxiety Disorder/Schizophrenia/Eating disorder/Alcohol use disorder/Other substance use disorder/Personality disorders/Other]* 2. *Please indicate which psychiatric disorder(s) you have ever been diagnosed with (multiple answers possible): [None/Depressive disorder/Bipolar disorder/Panic disorder/Generalized Anxiety Disorder/Schizophrenia/Eating disorder/Alcohol use disorder/Other substance use disorder/Personality disorders/Other]* 3. *Do you have first-degree relatives with a severe mental disorder (schizophrenia, bipolar disorder, major depressive disorder) [Yes/No]*

**Figure 1:**
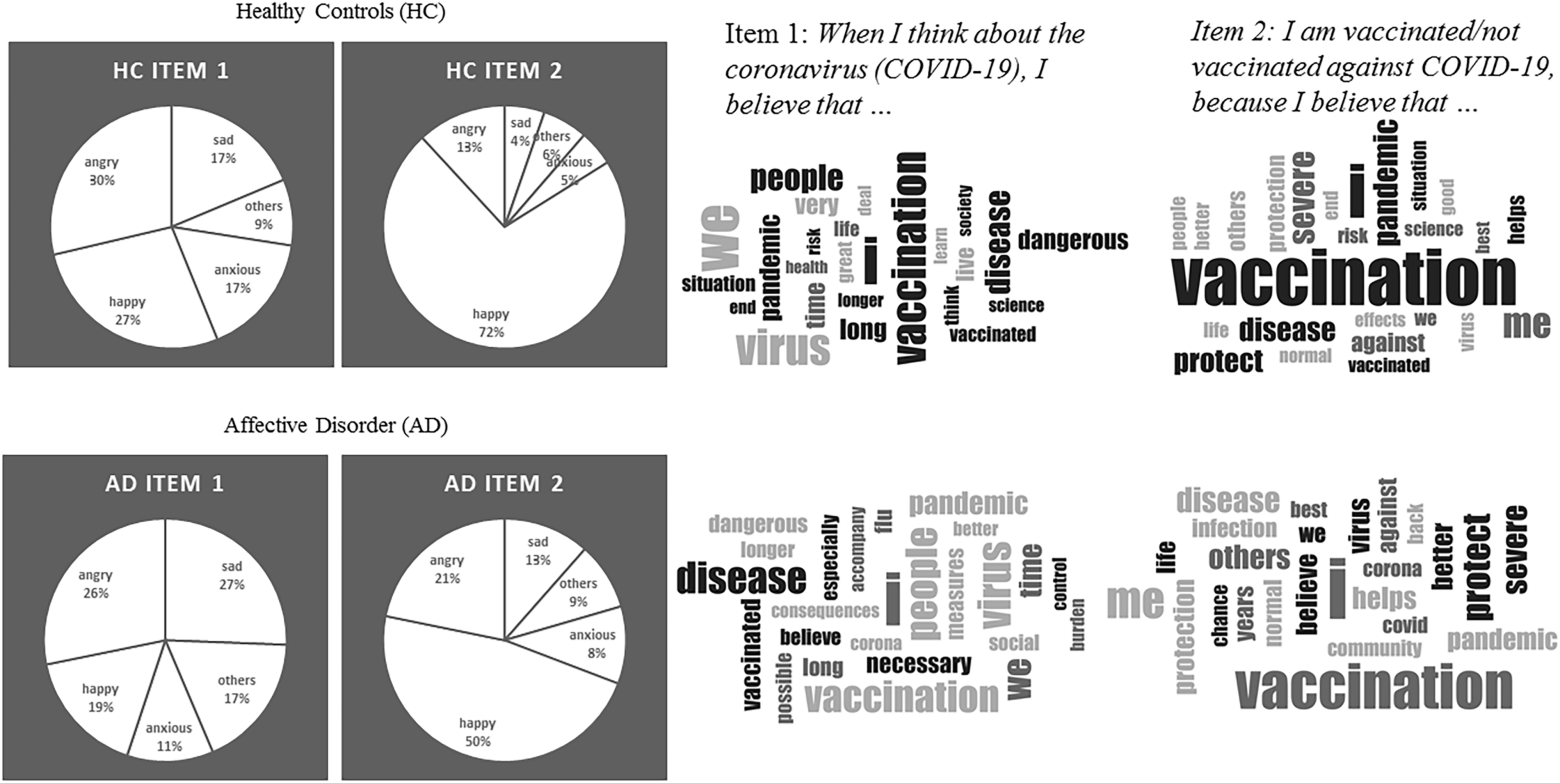
Frequencies in emotions and word clouds of items 1 and 2 in AD and HC.

In addition to the proposition (narrative), the degree of certainty, the experienced emotion while believing (evaluated *via* an Emotion Wheel, see Figure 1), and the mightiness (strength of emotion) were assessed. As certainty and mightiness were rating scales, emotion was categorized into positive (happy), negative (sad, angry, anxious, disgusted), and indifferent (surprised) emotions. In addition, it was evaluated whether the narrative was positive, negative, or indifferent, and whether it matched the emotion (congruent) or not (incongruent).

Although the survey was sent out to former patients of the Department of Psychiatry and Psychotherapeutic Medicine, some diagnoses of AD (unipolar or bipolar affective disorder) were self-reported, as the link was additionally shared *via* social media for volunteers (Facebook and WhatsApp). HC had to state no psychiatric disorder themselves or in first-degree relatives (see control items).

### Statistics

A multivariate analysis of co-variance (MANCOVA) with group (AD vs. HC) as independent variable controlling for age was calculated to test for between-subject differences in the credition parameters certainty and mightiness for both credition items. The *a priori* power analyses with Gpower 3.1.9.7. revealed for MANOVA (Global effects) a total sample size of *n* = 252, given effect size 0.0625, Power = 0.95, and Alpha = 0.05. As cell distribution was unequal in vaccination status, we could not enter this variable as second factor into the model but used *t*-tests to test for differences between vaccinated vs. non-vaccinated individuals (as homogeneity of variance was given). Differences in congruence (between emotion and narrative yes vs. no), emotion (positive = happy; negative = anxious, disgusted, angry, sad; indifferent = surprised), and narrative (positive, negative, indifferent) were calculated with chi-square tests and two-tailed Fisher’s exact tests when more than 20% of expected frequencies were > 5. MANCOVA assumptions (normal distribution, homogeneity of variance) were checked. In ANOVA models, partial eta square (η^2^_p_), and for *t*-tests, Cohen’s d as measure of effect size are presented. The obtained data were analyzed using IBM SPSS Statistics for Windows version 26.0 (Armonk, New York: IBM Corp). In addition, we created word clouds in MAXQDA 2020 (VERBI Software, 2019) to present propositions for each item in the groups. Prepositions and conjunctions were ignored and added to a stop list in MAXQDA. The word clouds were translated from German into English for this study.

## Results

In AD, the mean age was 43.78 years, 66.3% were female, and the median years of education were 16.20 years (see Table 2). In HC, the mean age was 37.17 years, 67.3% were female, and the median years of education were 16.7 years. Patients were significantly older than controls.

**Table 2:** Sociodemographic characteristics of individuals with affective disorder and healthy controls.

Variables	Group		Test statistic (*t*, *χ^2^*)	*p*-value	Cohen’s *d*
	AD (*n* = 79)	HC (*n* = 173)			
Age (*M* ± *SD*)	43.44 (13.86)	37.24 (13.36)	*t*(249) *= −3.38*	**<0.001**	−0.46
Sex (*n*, %)			χ^2^(1) = 0.05	0.818	
Female	52 (65.8%)	118 (67.3%)			
Male	27 (34.2%)	55 (31.8%)			
Median years of education (*M* ± *SD*)	16.25 (6.24)	16.66 (3.60)	*t*(102,469) = 0.55	0.586	0.09
Vaccination status (*n*, %)			*χ^2^*(1) = 2.75	0.098	
Vaccinated^a^ Unvaccinated	77 (97.5%) 2 (2.5%)	157 (90.8%) 16 (9.2%)			
Immunization against COVID-19 (*n*, %)			*χ^2^*(1) = 1.94	0.164	
Immunized^b^	76 (96.2%)	156 (90.2%)			
Not immunized	3 (3.8%)	17 (9.8%)			

AD = Affective disorder, HC = Healthy controls.

a At least one shot.

b At least two shots.

Bold value indicates a statistically significant difference *p*<0.05 between AD and HC.

In AD, 96.2%, and in HC, 90.4% were vaccinated, i.e., had received at least one vaccination. Regarding immunization, i.e., having received at least two vaccinations, 3.8% of patients with AD and 10.2% of HC were not vaccinated or had only received one vaccination.

### Differences between AD and HC

In response to COVID-19 beliefs in general (item 1), there was no multivariate effect [*F*(2,248) = 1.24, *p = 0*.291, η^2^_p =_ 0.01; see Table 3]. Chi-square tests showed that HC had more positive emotions and fewer indifferent emotions than individuals with AD, who showed more indifferent emotions. There was no difference between the groups in frequencies of congruence or content of narrative.

**Table 3:** Descriptive statistics of the believing parameters of individuals with affective disorders and healthy controls.

Variables	Group		Test statistic	*p*-value	*η_p_^2^*
	AD (*n* = 79)	HC (*n* = 173)			
**COVID-19 pandemic in general** ^a^					
Narratives (*n*, %)			*χ^2^*(2) = 5.17	0.075	
Positive	20 (25.3%)	57 (32.9%)			
Negative	57 (72.2%)	102 (59.0%)			
Indifferent	2 (2.5%)	14 (8.1%)			
Emotions (*n*, %)			χ^2^(2) = 8.78	**0.012**	
Positive	14 (17.7%)	47 (27.2%)			
Negative	52 (65.8%)	116 (67.1%)			
Indifferent	13 (16.5%)	10 (5.8%)			
Congruence^b^ (*n*, %)			χ^2^(1) = 0.43	0.440	
Congruent	56 (70.9%)	131 (75.7%)			
Incongruent	23 (29.1%)	42 (24.3%)			
Certainty^d^ (*M* ± *S)*	83.30 (15.32)	85.80 (16.64)	*F*(1,249) = 2.11	0.147	0.01
Mightiness^d^ (*M* ± *SD)*	66.04 (26.26)	69.47 (23.80)	*F*(1,249) = 0.93	0.337	0.00
**Vaccination** ^e^					
Narratives (*n*, %)			χ^2^*(*1) = 0.77^c^	0.771	
Positive	70 (88.6%)	152 (87.9%)			
Negative	8 (10.1%)	20 (11.6%)			
Indifferent	1 (1.3%)	1 (0.6%)			
Emotions (*n*, %)			χ^2^(2) = 9.97	**0.007**	
Positive	40 (50.6%)	123 (71.1%)			
Negative	34 (43.0%)	43 (24.9%)			
Indifferent	5 (6.3%)	7 (4.0%)			
Congruence^b^ (*n*, %)			χ^2^(1) = 3.84	0.071	
Congruent	53 (67.1%)	136 (78.6%)			
Incongruent	26 (32.9%)	37 (21.4%)			
Certainty^c^ (*M* ± *SD)*	85.58 (17.08)	91.79 (12.83)	*F*(1,249) = 10.38	**0.001**	0.04
Mightiness^c^ (*M* ± *SD)*	74.67 (23.94)	77.62 (19.76)	*F*(1,249) = 1.43	0.233	0.01

AD = Affective disorder, HC = Healthy controls.

a When I think about the coronavirus (COVID-19), I believe that…

b Congruence between the narratives and the emotions.

c Fisher’s exact test was used.

d In percent.

e I am vaccinated/not vaccinated against COVID-19, because I believe that….

Bold value indicates a statistically significant difference p<0.05 between AD and HC.

**Figure 2:**
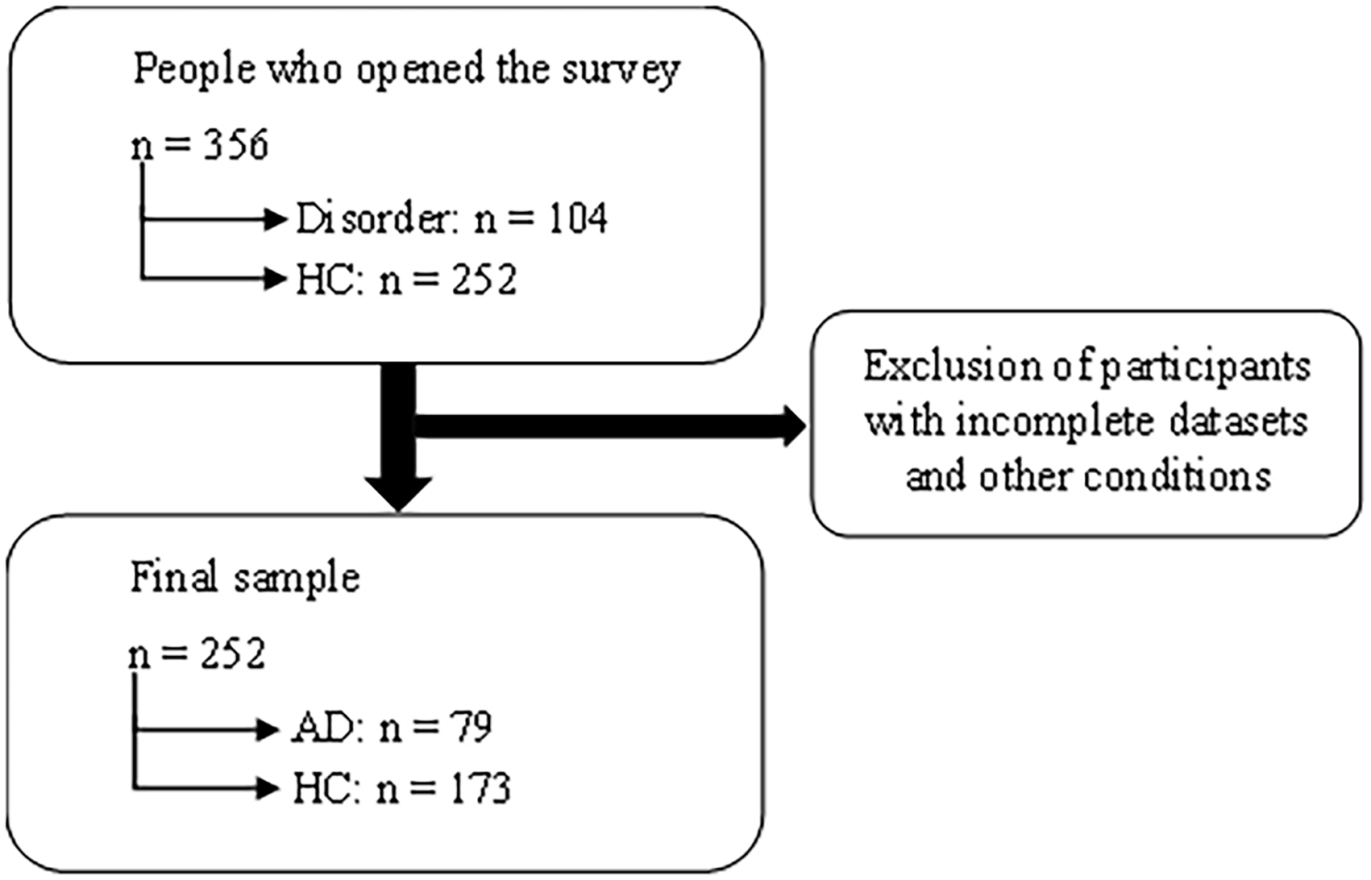
Flow chart of the participant selection.

Regarding item 2 Vaccination/Non-vaccination motive beliefs there was a significant multivariate group effect [*F*(2,248) = 5.19, *p = 0*.006, η^2^_p =_ 0.04; see Table 3] indicating higher certainty in HC than in AD. No group effects were shown in mightiness. In addition, emotion differed between AD and HC, the latter reporting more positive and less negative emotions (see Figure 2). Furthermore, there was more congruence between emotion and narrative in HC than in AD. No group differences were shown in content of narratives.

**Figure 3:**
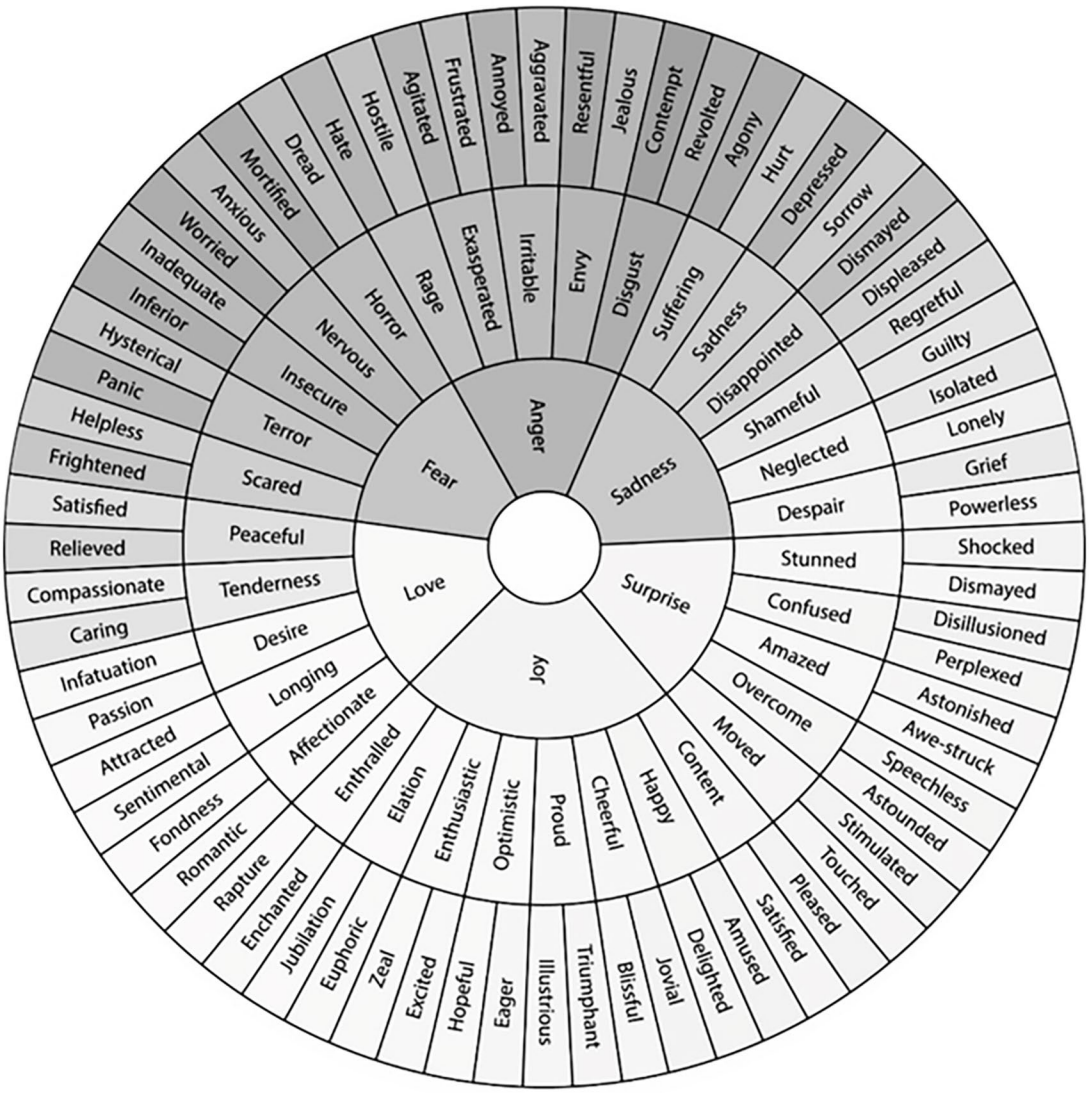
Emotion wheel used to select the emotion while believing.

Figure 3 shows the frequencies in emotions and the word clouds in AD vs. HC.

### Differences between vaccinated and non-vaccinated individuals

*T*-tests showed significantly higher certainty levels in non-vaccinated people (item 1: *M* = 90.6, *SD* = 15.7) than in vaccinated people [*M =* 83.9, *SD =* 16.4; *t*(255) = 1.75, *p = 0*.041, Cohen’s d = 0.35]. This effect was slightly higher for item 2 Vaccination/Non-vaccination motive beliefs [Non-vaccinated individuals: *M =* 94.1, *SD =* 9.9 vs. vaccinated individuals: *M =* 89.1, *SD =* 14.8; *t*(254) = 2.04, *p = 0*.026, Cohen’s d = 0.39]. No group differences in mightiness were observed item 1: *t*(255) = 0.05, *p* = 0.482; item 2: *t*(254) = 0.33, *p = 0*.370.

Vaccinated people showed highly more positive emotions (68.2%) when believing about their motives for vaccination in comparison to non-vaccinated individuals, who reported more negative emotions while believing in their motives for non-vaccination [63.2%; *χ^2^*(2) = 13.60, *p = 0*.001]. No group differences were found in emotion in item 1 [*χ^2^*(2) = 2.02, *p = 0*.364].

In addition, non-vaccinated people showed highly more negative (85% vs. 4.6%) and fewer positive narratives (10.0% vs. 93.7%) than vaccinated individuals [Fisher’s exact test: *χ^2^*(1) = 125.70, *p < 0*.001] for item 2. No group differences were found for item 1 [*χ^2^*(2) = 3.80, *p* = 0.149].

Two-tailed Fisher’s exact tests showed that frequencies of congruence did not differ between vaccinated and non-vaccinated people [item 1: *χ^2^*(1) = 1.31, *p* = 0.631; item 2: *χ^2^*(1) = 4.09, *p* = 0.165].

## Discussion

At the same time as the decision to introduce mandatory COVID-19 vaccinations in Austria in December 2021/January 2022, we surveyed 79 individuals with AD and 173 mentally healthy people. Their attitudes and beliefs about the coronavirus and their motives for vaccination/vs. non-vaccination were assessed using the parameters of the credition model (Angel, 2013; Angel and Seitz, 2016).

Individuals with AD and HC did not differ in their vaccination status. This has been shown in former Austrian studies with other samples (Fellendorf et al., 2022) as well as in international studies (Batty et al., 2021; Hao et al., 2021; Jefsen et al., 2021), although other studies showed a lower vaccine rate in individuals with psychiatric disorders (Arumuham et al., 2022; Curtis et al., 2022). We suppose a strong influence of socioeconomic circumstances, e.g., age, sex, education, and income, as well as cultural factors, such as governmental regulations in vaccination decision (Schwarzinger et al., 2021; Schernhammer et al., 2022). For example, both groups did not differ in education, although individuals with AD generally have lower levels of education (Lorant et al., 2003), which is more often found in unvaccinated individuals (Troiano and Nardi, 2021). There were also no differences in terms of sex. In this case, it would have been important to consider women’s lower vaccine uptake (Troiano and Nardi, 2021). In relation to government regulations, the lockdown for unvaccinated people as well as the upcoming obligatory vaccination could have strongly encouraged both Austrian HC and individuals with AD to get vaccinated. Moreover, there are no or only minimal private costs for healthcare in Austria for the individual, and although there were supply shortages, an easier general access to healthcare than in other countries could have further contributed to the results.

The COVID-19 pandemic is a highly emotional topic that is very much polarizing (Alam et al., 2021; Liew and Lee, 2021). This was also supported by the present study’s results. When thinking about the coronavirus, HC reported more positive and less indifferent emotions while believing than individuals with AD. This is consistent with other studies that found that individuals with psychiatric disorders experienced more distress during the pandemic than HC (Solé et al., 2021). However, two thirds of both individuals with AD and HC reported negative emotions (anger, sadness, anxiety) when believing about the coronavirus, highlighting the continued negative influence of the pandemic on the population even at the beginning of 2022.

When thinking about their motives of vaccination, individuals with AD reported more negative emotions while believing than HC, most of whom reported positive emotions. Comparably, other studies found less vaccine acceptance in individuals with mental illness (Danenberg et al., 2021; Huang et al., 2021; Payberah et al., 2022), which is linked to negative feelings about the vaccination (de Vries et al., 2022). However, as vaccination rate did not differ in this study, emotions supposedly might not have played the essential role for individuals with AD when deciding whether they wanted to get vaccinated.

The results further showed that individuals with AD were less certain about their beliefs, especially regarding the COVID-19 vaccination. We assume that patients with AD have developed greater insecurity about potential threats based on their existing chronic mental disease, which could also lead to more self-care or a more ambivalent/incongruent attitude according to the stress-vulnerability model. Other possible reasons for our results might be mistrust, misinformation, and heightened fear, which has been shown to relate to vaccination hesitancy in individuals with mental illness (Payberah et al., 2022; Peritogiannis et al., 2022). The finding that individuals with a psychiatric disorder show less certainty about what they believe has also been observed in our first credition study in a sample of bipolar disorder (Tietz et al., 2022).

Independently from diagnosis, lower levels of certainty were also observed in vaccinated compared to non-vaccinated individuals. We assume that someone who is not vaccinated decides so with greater conviction (than someone who is vaccinated), and very strong negative emotions go along with it as supported by our findings. This goes in line with results by de Vries et al. (2022) demonstrating that individuals with vaccine hesitancy were less convinced.

of the emotional and rational advantages of COVID-19 vaccination and expressed more negative feelings about it. However, underlying reasons for non-vaccinations, including beliefs, have to be explored in samples with larger sample sizes.

This study has the following limitations. One problem of online studies is the sampling bias, such that only data from individuals who were motivated to participate in the survey were collected. This explains why most participants were vaccinated at least once and the group of unvaccinated was rather small. As vaccination rate in Austria was 70% at this time, there was a higher likelihood to recruit vaccinated people in a random sample (Epidemiologisches Meldesystem, 2022a). Thus, the cell sizes between vaccinated and unvaccinated individuals were too small to perform further statistical calculations, e.g., a 2 × 2 design with group and vaccination status would have been desirable. In addition, the diagnoses of AD were self-reported, but several control items were included. Moreover, instead of believing processes themselves, only verbal expressions could be examined. Believing processes might have been influenced by the subjects’ introspective ability, which was not measured in the study. Furthermore, qualitative data had to be reduced by transforming into positive, negative, and indifferent. It should also be noted that information may have been lost because of translation.

In conclusion, people with AD were more uncertain and experienced fewer positive emotions when thinking about their beliefs in the COVID-19 vaccination than HC. However, as both groups did not differ in vaccination rate, sociopolitical circumstances were presumably more influential in the decision to get vaccinated. Unvaccinated people were more likely to display negative emotions and narratives accompanied by high levels of certainty while believing in their motives for not getting vaccinated, but not when believing in the coronavirus in general; however, the cases of unvaccinated individuals were too small to draw final conclusions.

## Data availability statement

The raw data supporting the conclusions of this article will be made available by the authors, without undue reservation.

## Ethics statement

The studies involving human participants were reviewed and approved by Medical University of Graz, Austria. The patients/participants provided their written informed consent to participate in this study.

## Author contributions

ND designed the study. ND and EF performed literature research as well as data analysis and wrote the first draft. FF, JW-S, ES, SB, AH, ST, AT-B, ML, and ER were responsible for proof reading and revising the manuscript. ES additionally supported the implementation of the study *via* the online application tool LimeSurvey. ER supervised the study procedure and revised important intellectual content. All authors contributed to the article and approved the submitted version.

## Funding

The authors declare that this study received funding from the Volkswagen Foundation and the Betz Foundation. The authors declare that this study received funding from Siemens Healthineers. The funder was not involved in the study design, collection, analysis, interpretation of data, the writing of this article, or the decision to submit it for publication.

## Conflict of interest

The authors declare that the research was conducted in the absence of any commercial or financial relationships that could be construed as a potential conflict of interest.

## Publisher’s note

All claims expressed in this article are solely those of the authors and do not necessarily represent those of their affiliated organizations, or those of the publisher, the editors and the reviewers. Any product that may be evaluated in this article, or claim that may be made by its manufacturer, is not guaranteed or endorsed by the publisher.
